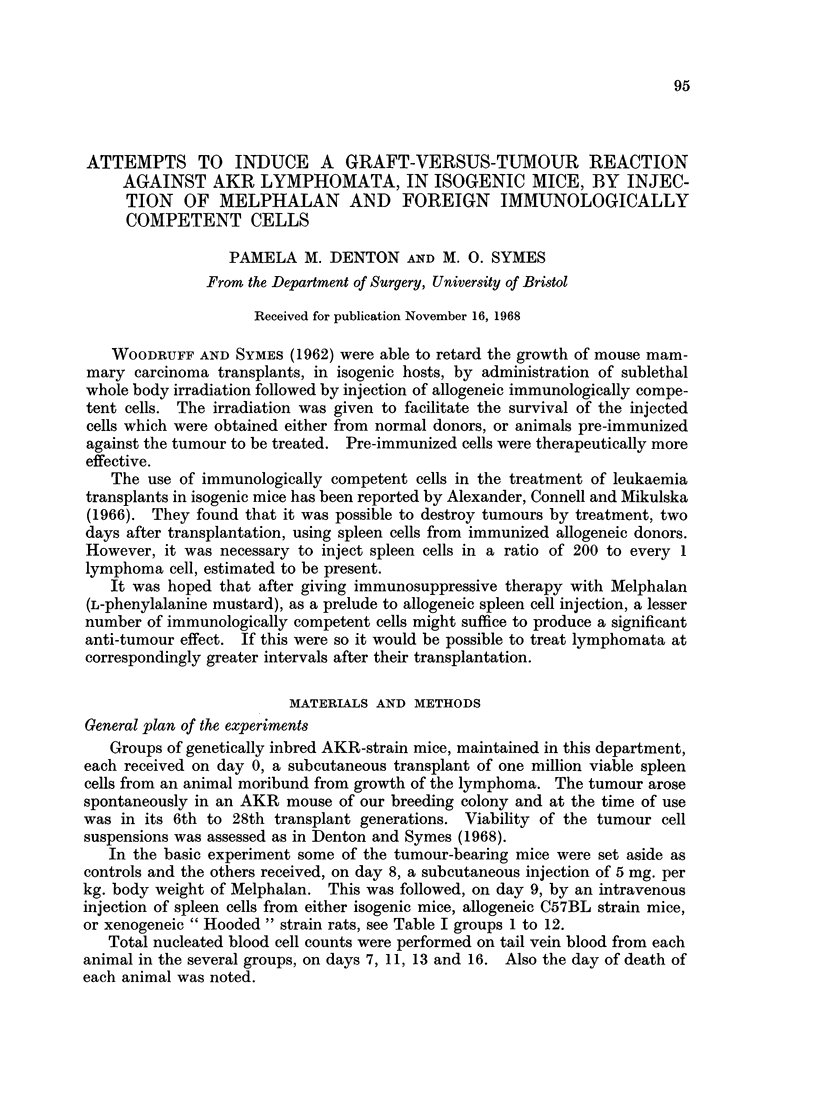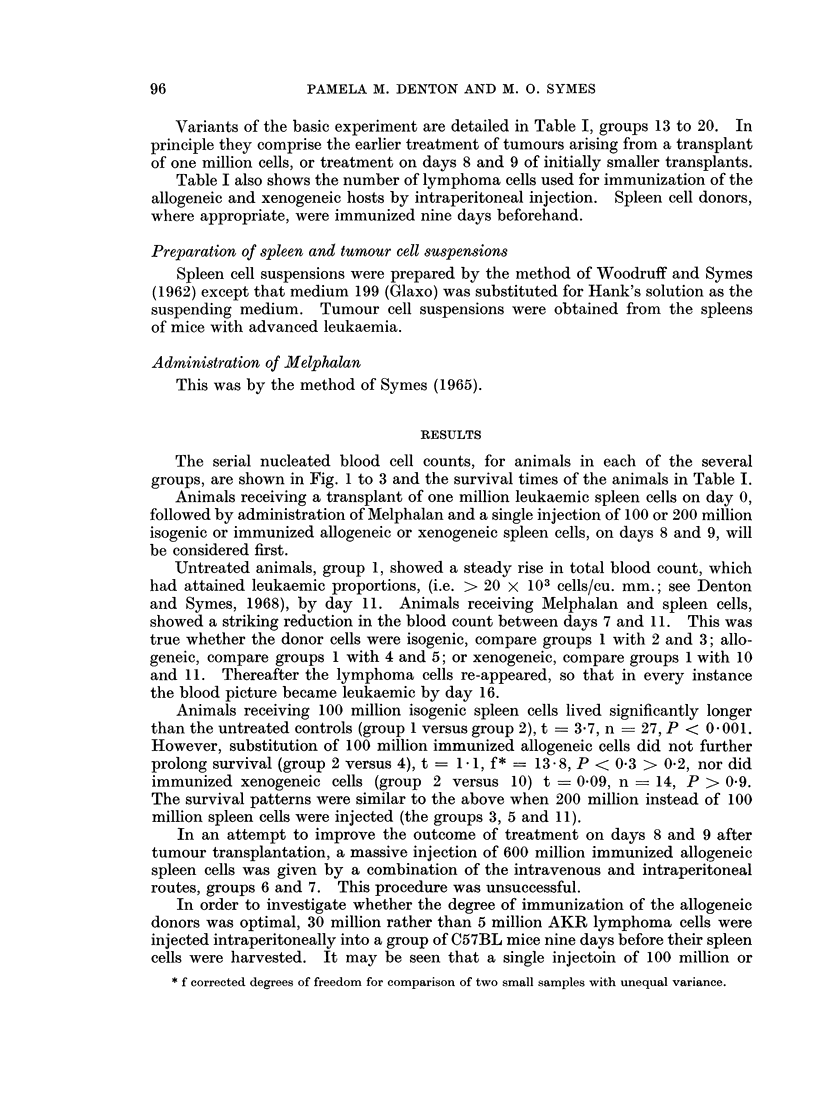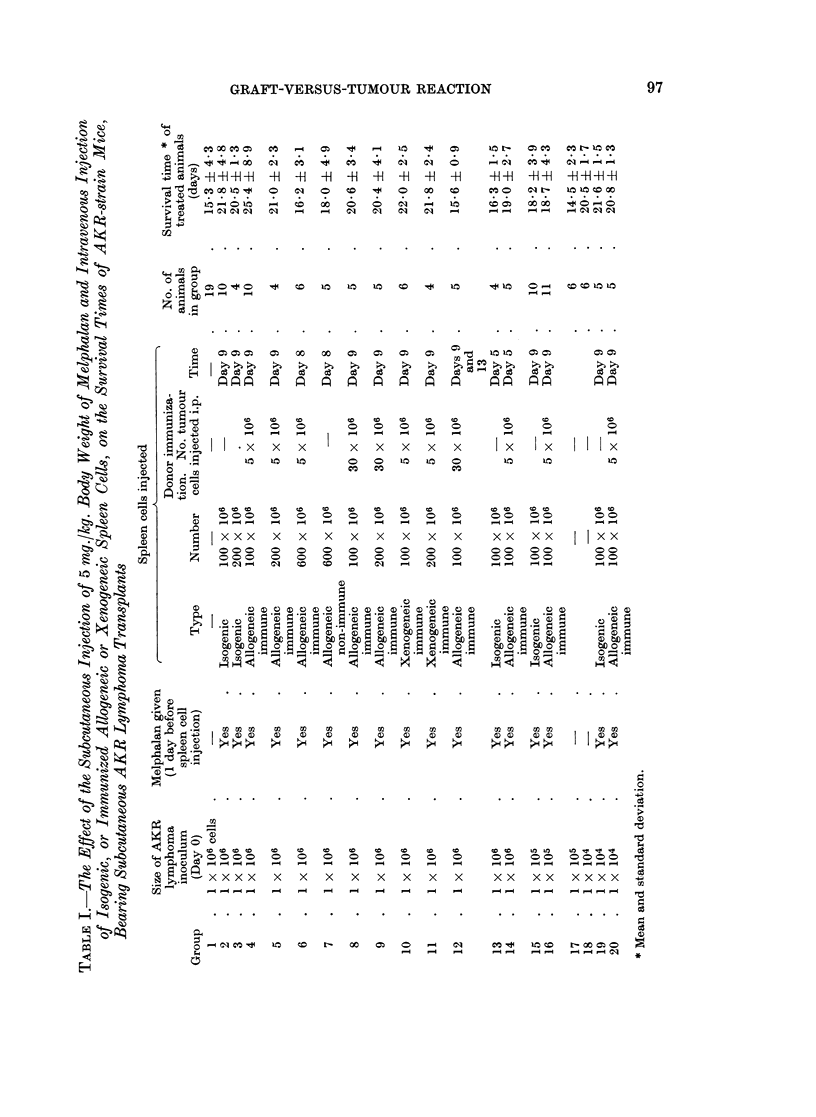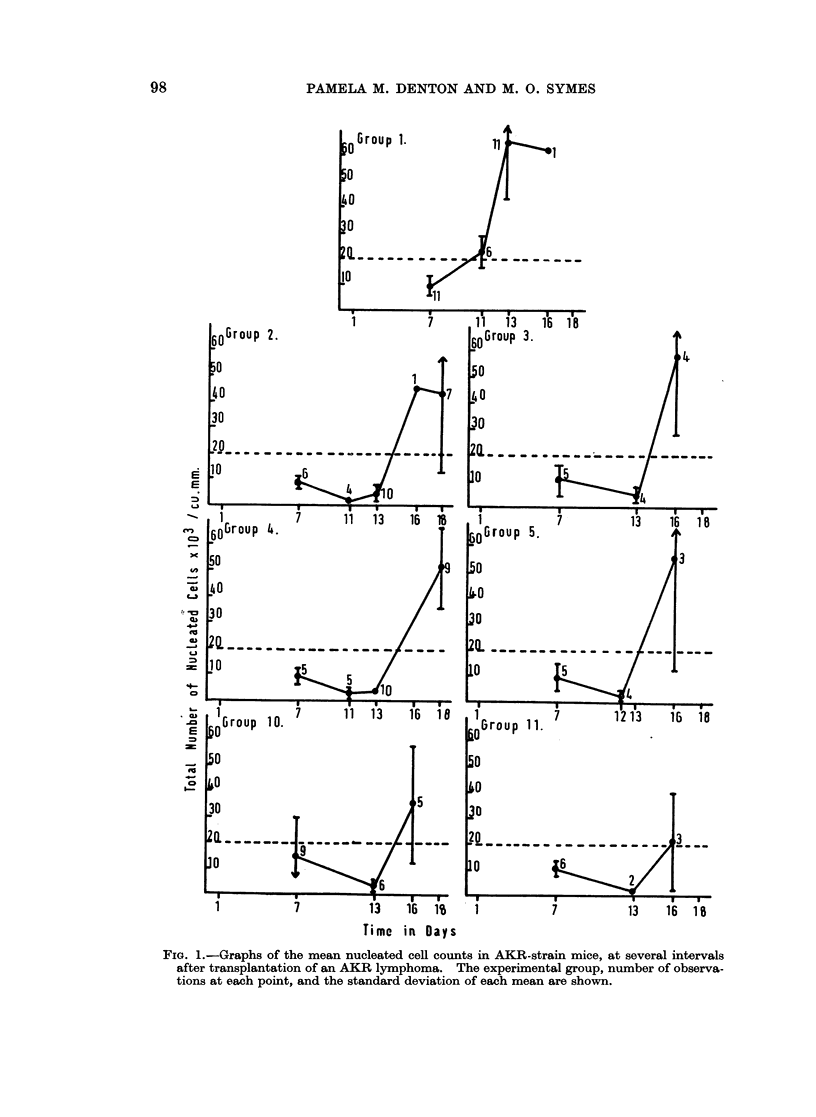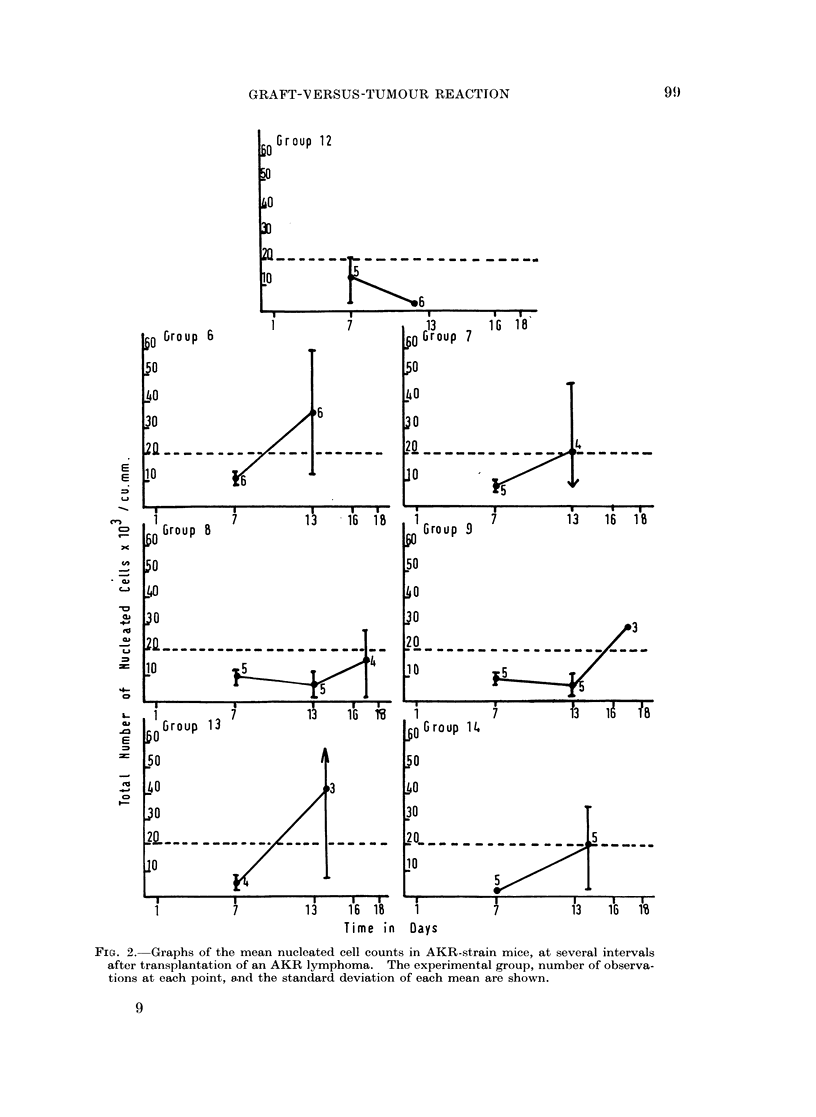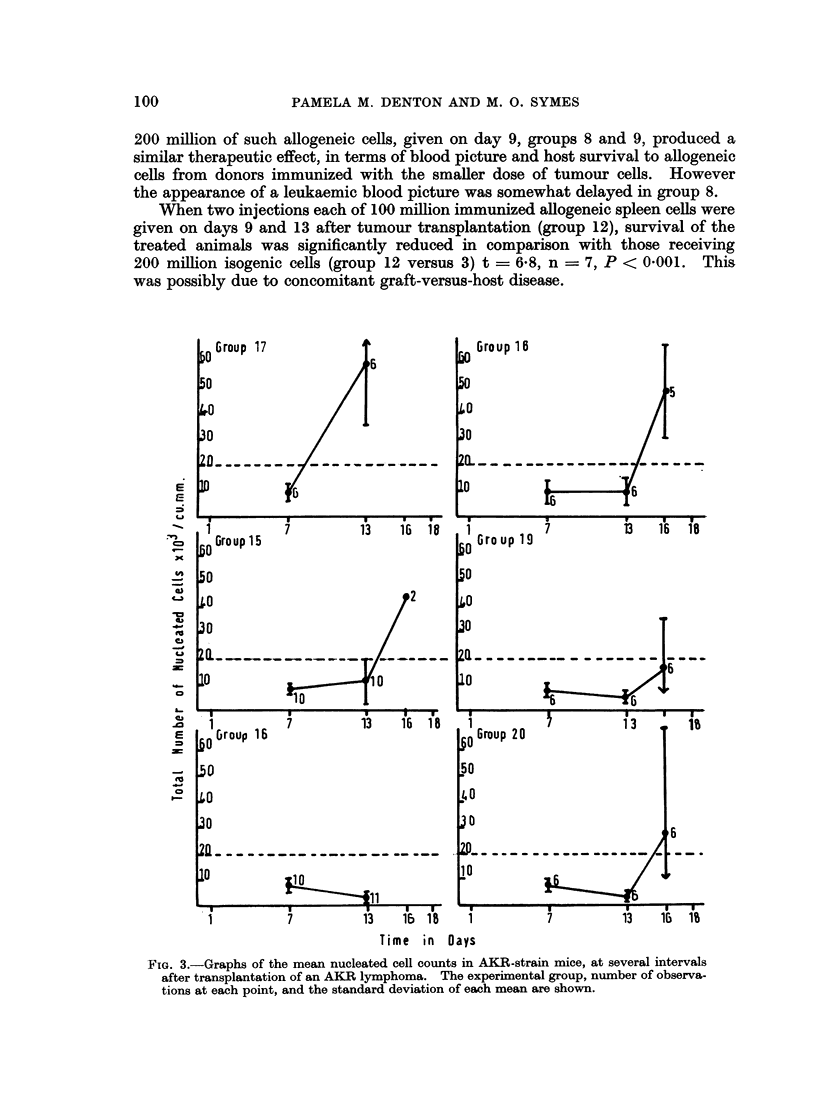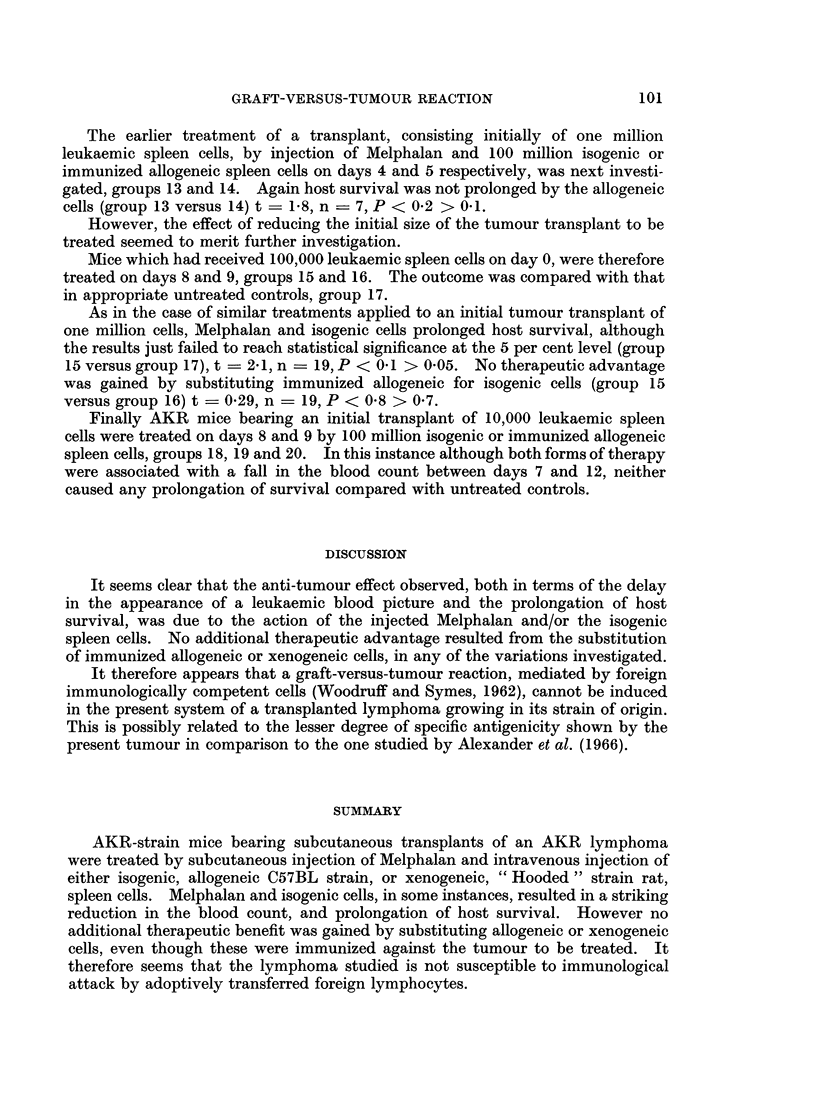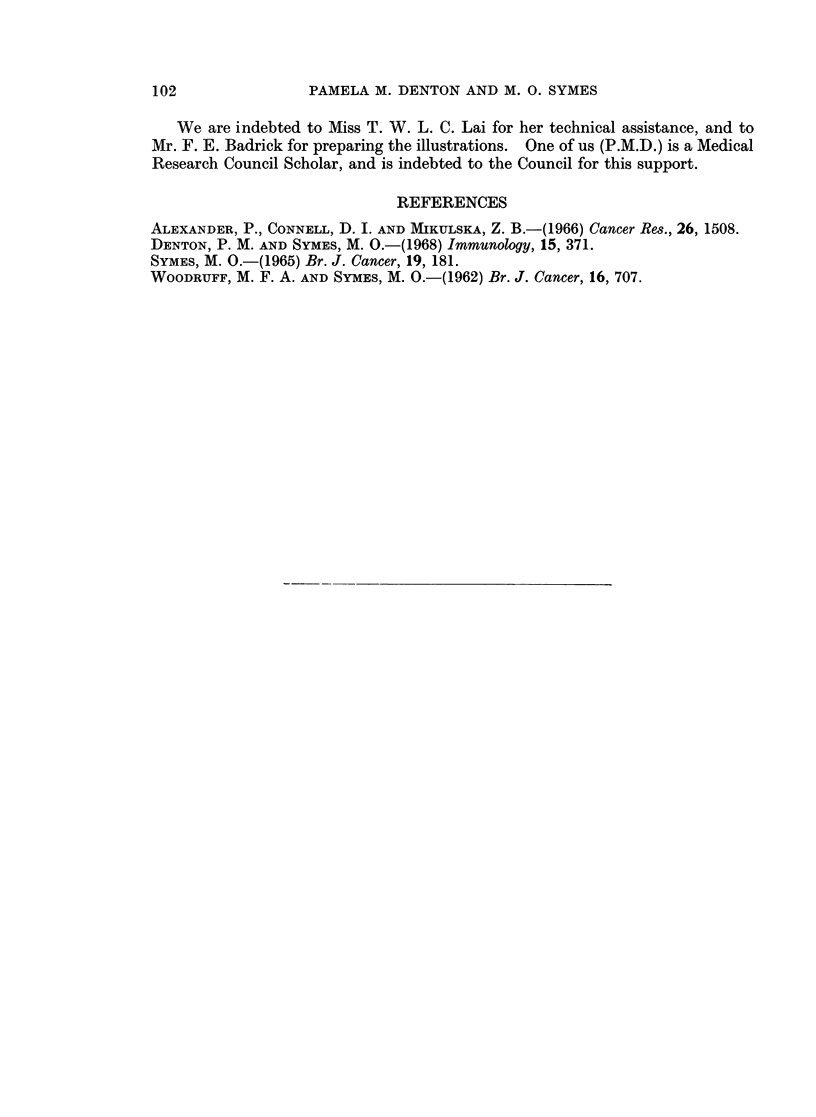# Attempts to induce a graft-versus-tumour reaction against AKR lymphomata, in isogenic mice, by injection of Melphalan and foreign immunologically competent cells.

**DOI:** 10.1038/bjc.1969.15

**Published:** 1969-03

**Authors:** P. M. Denton, M. O. Symes


### ATTEMPTS TO INDUCE A GRAFT-VERSUS-TUMOUR REACTION AGAINST AKR LYMPHOMATA, IN ISOGENIC MICE, BY INJECTION OF MELPHALAN AND FOREIGN IMMUNOLOGICALLY COMPETENT CELLS

PAMELA M. DENTON AND M. O. SYMES

*From the Department of Surgery, University of Bristol*

Received for publication November 16, 1968

WOODRUFF AND SYMES (1962) were able to retard the growth of mouse mammary carcinoma transplants, in isogenic hosts, by administration of sublethal whole body irradiation followed by injection of allogeneic immunologically competent cells. The irradiation was given to facilitate the survival of the injected cells which were obtained either from normal donors, or animals pre-immunized against the tumour to be treated. Pre-immunized cells were therapeutically more effective.

The use of immunologically competent cells in the treatment of leukaemia transplants in isogenic mice has been reported by Alexander, Connell and Mikulska (1966). They found that it was possible to destroy tumours by treatment, two days after transplantation, using spleen cells from immunized allogeneic donors. However, it was necessary to inject spleen cells in a ratio of 200 to every 1 lymphoma cell, estimated to be present.

It was hoped that after giving immunosuppressive therapy with Melphalan (L-phenylalanine mustard), as a prelude to allogeneic spleen cell injection, a lesser number of immunologically competent cells might suffice to produce a significant anti-tumour effect. If this were so it would be possible to treat lymphomata at correspondingly greater intervals after their transplantation.

### MATERIALS AND METHODS

### *General plan of the experiments*

Groups of genetically inbred AKR-strain mice, maintained in this department, each received on day 0, a subcutaneous transplant of one million viable spleen cells from an animal moribund from growth of the lymphoma. The tumour arose spontaneously in an AKR mouse of our breeding colony and at the time of use was in its 6th to 28th transplant generations. Viability of the tumour cell suspensions was assessed as in Denton and Symes (1968).

In the basic experiment some of the tumour-bearing mice were set aside as controls and the others received, on day 8, a subcutaneous injection of 5 mg. per kg. body weight of Melphalan. This was followed, on day 9, by an intravenous injection of spleen cells from either isogenic mice, allogeneic C57BL strain mice, or xenogeneic " Hooded " strain rats, see Table I groups 1 to 12.

Total nucleated blood cell counts were performed on tail vein blood from each animal in the several groups, on days 7, 11, 13 and 16. Also the day of death of each animal was noted.

Variants of the basic experiment are detailed in Table I, groups 13 to 20. In principle they comprise the earlier treatment of tumours arising from a transplant of one million cells, or treatment on days 8 and 9 of initially smaller transplants.

Table I also shows the number of lymphoma cells used for immunization of the allogeneic and xenogeneic hosts by intraperitoneal injection. Spleen cell donors, where appropriate, were immunized nine days beforehand.

*Preparation of spleen and tumour cell suspensions*

Spleen cell suspensions were prepared by the method of Woodruff and Symes (1962) except that medium 199 (Glaxo) was substituted for Hank's solution as the suspending medium. Tumour cell suspensions were obtained from the spleens of mice with advanced leukaemia.

*Administration of Melphalan*

This was by the method of Symes (1965).

### RESULTS

The serial nucleated blood cell counts, for animals in each of the several groups, are shown in Fig. 1 to 3 and the survival times of the animals in Table I.

Animals receiving a transplant of one million leukaemic spleen cells on day 0, followed by administration of Melphalan and a single injection of 100 or 200 million isogenic or immunized allogeneic or xenogeneic spleen cells, on days 8 and 9, will be considered first.

Untreated animals, group 1, showed a steady rise in total blood count, which had attained leukaemic proportions, (i.e. $> 20 \times 10^3$ cells/cu. mm.; see Denton and Symes, 1968), by day 11. Animals receiving Melphalan and spleen cells, showed a striking reduction in the blood count between days 7 and 11. This was true whether the donor cells were isogenic, compare groups 1 with 2 and 3; allogeneic, compare groups 1 with 4 and 5; or xenogeneic, compare groups 1 with 10 and 11. Thereafter the lymphoma cells re-appeared, so that in every instance the blood picture became leukaemic by day 16.

Animals receiving 100 million isogenic spleen cells lived significantly longer than the untreated controls (group 1 versus group 2), $t = 3{\cdot}7$, $n = 27$, $P < 0{\cdot}001$. However, substitution of 100 million immunized allogeneic cells did not further prolong survival (group 2 versus 4), $t = 1{\cdot}1$, $f^* = 13{\cdot}8$, $P < 0{\cdot}3 > 0{\cdot}2$, nor did immunized xenogeneic cells (group 2 versus 10) $t = 0{\cdot}09$, $n = 14$, $P > 0{\cdot}9$. The survival patterns were similar to the above when 200 million instead of 100 million spleen cells were injected (the groups 3, 5 and 11).

In an attempt to improve the outcome of treatment on days 8 and 9 after tumour transplantation, a massive injection of 600 million immunized allogeneic spleen cells was given by a combination of the intravenous and intraperitoneal routes, groups 6 and 7. This procedure was unsuccessful.

In order to investigate whether the degree of immunization of the allogeneic donors was optimal, 30 million rather than 5 million AKR lymphoma cells were injected intraperitoneally into a group of C57BL mice nine days before their spleen cells were harvested. It may be seen that a single injectoin of 100 million or

* f corrected degrees of freedom for comparison of two small samples with unequal variance.

TABLE I.—*The Effect of the Subcutaneous Injection of 5 mg./kg. Body Weight of Melphalan and Intravenous Injection of Isogenic, or Immunized Allogeneic or Xenogeneic Spleen Cells, on the Survival Times of AKR-strain Mice, Bearing Subcutaneous AKR Lymphoma Transplants*

| Group | Size of AKR lymphoma inoculum (Day 0) | Melphalan given (1 day before spleen cell injection) | Spleen cells injected: Type | Spleen cells injected: Number | Spleen cells injected: Donor immunization. No. tumour cells injected i.p. | Spleen cells injected: Time | No. of animals in group | Survival time * of treated animals (days) |
|---|---|---|---|---|---|---|---|---|
| 1 | 1 × $10^6$ cells | — | — | — | — | — | 19 | 15·3 ± 4·3 |
| 2 | 1 × $10^6$ | Yes | Isogenic | 100 × $10^6$ | — | Day 9 | 10 | 21·8 ± 4·8 |
| 3 | 1 × $10^6$ | Yes | Isogenic | 200 × $10^6$ | . | Day 9 | 4 | 20·5 ± 1·3 |
| 4 | 1 × $10^6$ | Yes | Allogeneic immune | 100 × $10^6$ | 5 × $10^6$ | Day 9 | 10 | 25·4 ± 8·9 |
| 5 | 1 × $10^6$ | Yes | Allogeneic immune | 200 × $10^6$ | 5 × $10^6$ | Day 9 | 4 | 21·0 ± 2·3 |
| 6 | 1 × $10^6$ | Yes | Allogeneic immune | 600 × $10^6$ | 5 × $10^6$ | Day 8 | 6 | 16·2 ± 3·1 |
| 7 | 1 × $10^6$ | Yes | Allogeneic non-immune | 600 × $10^6$ | — | Day 8 | 5 | 18·0 ± 4·9 |
| 8 | 1 × $10^6$ | Yes | Allogeneic immune | 100 × $10^6$ | 30 × $10^6$ | Day 9 | 5 | 20·6 ± 3·4 |
| 9 | 1 × $10^6$ | Yes | Allogeneic immune | 200 × $10^6$ | 30 × $10^6$ | Day 9 | 5 | 20·4 ± 4·1 |
| 10 | 1 × $10^6$ | Yes | Xenogeneic immune | 100 × $10^6$ | 5 × $10^6$ | Day 9 | 6 | 22·0 ± 2·5 |
| 11 | 1 × $10^6$ | Yes | Xenogeneic immune | 200 × $10^6$ | 5 × $10^6$ | Day 9 | 4 | 21·8 ± 2·4 |
| 12 | 1 × $10^6$ | Yes | Allogeneic immune | 100 × $10^6$ | 30 × $10^6$ | Days 9 and 13 | 5 | 15·6 ± 0·9 |
| 13 | 1 × $10^6$ | Yes | Isogenic | 100 × $10^6$ | — | Day 5 | 4 | 16·3 ± 1·5 |
| 14 | 1 × $10^6$ | Yes | Allogeneic immune | 100 × $10^6$ | 5 × $10^6$ | Day 5 | 5 | 19·0 ± 2·7 |
| 15 | 1 × $10^5$ | Yes | Isogenic | 100 × $10^6$ | — | Day 9 | 10 | 18·2 ± 3·9 |
| 16 | 1 × $10^5$ | Yes | Allogeneic immune | 100 × $10^6$ | 5 × $10^6$ | Day 9 | 11 | 18·7 ± 4·3 |
| 17 | 1 × $10^5$ | — | | — | — | | 6 | 14·5 ± 2·3 |
| 18 | 1 × $10^4$ | — | | — | — | | 6 | 20·5 ± 1·7 |
| 19 | 1 × $10^4$ | Yes | Isogenic | 100 × $10^6$ | — | Day 9 | 5 | 21·6 ± 1·5 |
| 20 | 1 × $10^4$ | Yes | Allogeneic immune | 100 × $10^6$ | 5 × $10^6$ | Day 9 | 5 | 20·8 ± 1·3 |

* Mean and standard deviation.

FIG. 1.—Graphs of the mean nucleated cell counts in AKR-strain mice, at several intervals after transplantation of an AKR lymphoma. The experimental group, number of observations at each point, and the standard deviation of each mean are shown.

FIG. 2.—Graphs of the mean nucleated cell counts in AKR-strain mice, at several intervals after transplantation of an AKR lymphoma. The experimental group, number of observations at each point, and the standard deviation of each mean are shown.

200 million of such allogeneic cells, given on day 9, groups 8 and 9, produced a similar therapeutic effect, in terms of blood picture and host survival to allogeneic cells from donors immunized with the smaller dose of tumour cells. However the appearance of a leukaemic blood picture was somewhat delayed in group 8.

When two injections each of 100 million immunized allogeneic spleen cells were given on days 9 and 13 after tumour transplantation (group 12), survival of the treated animals was significantly reduced in comparison with those receiving 200 million isogenic cells (group 12 versus 3) t = 6·8, n = 7, $P < 0{\cdot}001$. This was possibly due to concomitant graft-versus-host disease.

FIG. 3.—Graphs of the mean nucleated cell counts in AKR-strain mice, at several intervals after transplantation of an AKR lymphoma. The experimental group, number of observations at each point, and the standard deviation of each mean are shown.

The earlier treatment of a transplant, consisting initially of one million leukaemic spleen cells, by injection of Melphalan and 100 million isogenic or immunized allogeneic spleen cells on days 4 and 5 respectively, was next investigated, groups 13 and 14. Again host survival was not prolonged by the allogeneic cells (group 13 versus 14) $t = 1{\cdot}8$, $n = 7$, $P < 0{\cdot}2 > 0{\cdot}1$.

However, the effect of reducing the initial size of the tumour transplant to be treated seemed to merit further investigation.

Mice which had received 100,000 leukaemic spleen cells on day 0, were therefore treated on days 8 and 9, groups 15 and 16. The outcome was compared with that in appropriate untreated controls, group 17.

As in the case of similar treatments applied to an initial tumour transplant of one million cells, Melphalan and isogenic cells prolonged host survival, although the results just failed to reach statistical significance at the 5 per cent level (group 15 versus group 17), $t = 2{\cdot}1$, $n = 19$, $P < 0{\cdot}1 > 0{\cdot}05$. No therapeutic advantage was gained by substituting immunized allogeneic for isogenic cells (group 15 versus group 16) $t = 0{\cdot}29$, $n = 19$, $P < 0{\cdot}8 > 0{\cdot}7$.

Finally AKR mice bearing an initial transplant of 10,000 leukaemic spleen cells were treated on days 8 and 9 by 100 million isogenic or immunized allogeneic spleen cells, groups 18, 19 and 20. In this instance although both forms of therapy were associated with a fall in the blood count between days 7 and 12, neither caused any prolongation of survival compared with untreated controls.

### DISCUSSION

It seems clear that the anti-tumour effect observed, both in terms of the delay in the appearance of a leukaemic blood picture and the prolongation of host survival, was due to the action of the injected Melphalan and/or the isogenic spleen cells. No additional therapeutic advantage resulted from the substitution of immunized allogeneic or xenogeneic cells, in any of the variations investigated.

It therefore appears that a graft-versus-tumour reaction, mediated by foreign immunologically competent cells (Woodruff and Symes, 1962), cannot be induced in the present system of a transplanted lymphoma growing in its strain of origin. This is possibly related to the lesser degree of specific antigenicity shown by the present tumour in comparison to the one studied by Alexander *et al.* (1966).

### SUMMARY

AKR-strain mice bearing subcutaneous transplants of an AKR lymphoma were treated by subcutaneous injection of Melphalan and intravenous injection of either isogenic, allogeneic C57BL strain, or xenogeneic, " Hooded " strain rat, spleen cells. Melphalan and isogenic cells, in some instances, resulted in a striking reduction in the blood count, and prolongation of host survival. However no additional therapeutic benefit was gained by substituting allogeneic or xenogeneic cells, even though these were immunized against the tumour to be treated. It therefore seems that the lymphoma studied is not susceptible to immunological attack by adoptively transferred foreign lymphocytes.

We are indebted to Miss T. W. L. C. Lai for her technical assistance, and to Mr. F. E. Badrick for preparing the illustrations. One of us (P.M.D.) is a Medical Research Council Scholar, and is indebted to the Council for this support.

### REFERENCES

ALEXANDER, P., CONNELL, D. I. AND MIKULSKA, Z. B.—(1966) *Cancer Res.*, **26**, 1508.
DENTON, P. M. AND SYMES, M. O.—(1968) *Immunology*, **15**, 371.
SYMES, M. O.—(1965) *Br. J. Cancer*, **19**, 181.
WOODRUFF, M. F. A. AND SYMES, M. O.—(1962) *Br. J. Cancer*, **16**, 707.